# Maternal vitamin D status and preterm birth: an eight-year retrospective cohort study in the Southeastern United States

**DOI:** 10.1038/s41372-026-02757-z

**Published:** 2026-06-22

**Authors:** Anjali G. Borsum, Maya F. Andrade, Myla D. Ebeling, Jeffrey E. Korte, Donna D. Johnson, Roger B. Newman, Bruce W. Hollis, Carol L. Wagner

**Affiliations:** 1 College of Medicine, Medical University of South Carolina, Charleston, SC USA; 2 Division of Neonatology, Department of Pediatrics, Shawn Jenkins Children’s Hospital, Medical University of South Carolina, Charleston, SC USA; 3 Department of Public Health Sciences, Medical University of South Carolina, Charleston, SC USA; 4 Division of Maternal Fetal Medicine, Department of Obstetrics and Gynecology, Medical University of South Carolina, Charleston, SC USA

**Keywords:** Public health, Calcium and vitamin D

## Abstract

**Objective:**

Investigate the association between vitamin D status (as measured by 25-hydroxyvitamin D concentration [25(OH)D]) and gestational age at delivery.

**Study design:**

This was a retrospective chart review of electronic medical records (*n* = 15,506) from women delivering at the Medical University of South Carolina, with first maternal 25(OH)D concentrations measured from January 2016 to March 2024.

**Result:**

Women delivering <37 weeks (*n* = 1652; 31.3 ± 17.8 ng/mL) had lower 25(OH)D concentrations compared to those delivering at 37 weeks or later (*n* = 13,451; 34.8 ± 17.3 ng/mL), with a mean difference of –3.46 ng/mL (95% Confidence Interval (CI): –4.54 to –2.38, *p* < 0.0001). This difference was even more pronounced among women delivering before 32 weeks (*n* = 385; 26.2 ± 14.9 ng/mL), who had substantially lower 25(OH)D concentrations than term deliveries, with a mean difference of –8.56 ng/mL (95% CI: –10.70 to –6.42, *p* < 0.0001).

**Conclusion:**

Maternal vitamin D status was associated with gestational age at delivery. Lower maternal 25(OH)D concentrations were observed among women who delivered preterm compared with those delivering at term. These findings suggest a potential relationship between vitamin D status and preterm birth and support further research into whether optimizing vitamin D status during pregnancy may improve perinatal outcomes.

## Introduction

Preterm birth (PTB), or birth before 37 weeks gestation, is an essential cause of neonatal morbidity and mortality. Globally, around 10% of births are premature and cause ~35% of neonatal deaths [1]. Prematurity is the leading cause of death of children under five worldwide [1]. Complications of prematurity include respiratory distress syndrome, necrotizing enterocolitis, patent ductus arteriosus, intraventricular hemorrhage, feeding intolerance, and sepsis in the neonatal period, as well as longer-term sequelae such as bronchopulmonary dysplasia, retinopathy of prematurity, and neurodevelopmental impairment [2]. Very preterm birth is defined as birth before 32 weeks gestation and is associated with even higher risks and severity of adverse outcomes [3, 4]. Infants born before 32 weeks have higher rates of morbidity and mortality, longer rates of hospitalization, and more severe long-term impairment [5, 6]. Each additional week of gestation is associated with improved fetal outcomes [3, 4]; thus, optimizing maternal health to extend pregnancy to term safely is critical.

Preterm birth arises from a range of maternal, fetal, and environmental factors, including intrauterine infection or inflammation, hypertensive disorders, multiple gestation, uteroplacental insufficiency, and nutritional deficiencies [7]. Among nutritional factors, vitamin D (cholecalciferol [vitamin D₃] or ergocalciferol [vitamin D₂]), as a secosteroid and preprohormone, is a fat-soluble vitamin that plays a critical role in maintaining various physiological processes in both mother and child, including calcium homeostasis, immune modulation relevant to placental function and inflammatory pathways, and bone health [8–11]. Its significance extends beyond these established functions, particularly during pregnancy, where adequate levels are essential for maternal and fetal well-being, including proper skeletal development, immune function, and cell growth regulation [8]. Early in pregnancy, circulating 1,25-dihydroxyvitamin D (1,25(OH)₂D) increases two- to threefold relative to pre-pregnancy blood concentrations, underscoring both increased physiological demand and greater susceptibility to deficiency. The first metabolite of vitamin D is 25(OH)D, and with its longer circulating half-life of 2-3 weeks, it is the biomarker of vitamin D status [8].

While most agree that the minimum 25(OH)D concentration required to maintain a healthy metabolism in both mother and fetus during pregnancy is 30 ng/mL [12], other studies have shown that the optimal level of 25(OH)D during pregnancy is around 40 ng/mL [8, 13]. This is the point where the rate of conversion to 1,25(OH)_2_D is the highest [8]. Vitamin D insufficiency (less than 30 ng/mL) and deficiency (less than 20 ng/mL) are prevalent public health problems in the United States. It is estimated that the rates of vitamin D deficiency and insufficiency were 28.9% and 41.4%, respectively, in the US from 2001 to 2010, and certain populations, including pregnant women, are even more at risk [14]. In a study that examined vitamin D status in pregnant women in South Carolina, reflecting a diverse patient population that included Black American and Hispanic women, 82% were either insufficient or deficient [15, 16]. Severe vitamin D deficiency during pregnancy is defined as less than 10 ng/mL [17–19]. While many studies have linked severe deficiency with serious maternal and fetal adverse outcomes [9, 17, 19–21], those studies involved small sample sizes.

As previously stated, pregnant women require sufficient vitamin D, either through adequate sunlight exposure or supplementation, to achieve a target 25(OH)D concentration of at least 40 ng/mL [13, 22]. The National Institute of Medicine and the Endocrine Society suggest that 400–600 IU of vitamin D daily is sufficient to prevent deficiency in pregnant women, but up to 5000 IU/day has been shown to be safe [17]. Some studies, in fact, have shown that supplementation with 4000–4400 IU/day for pregnant women has been most efficacious in achieving sufficiency, with no adverse events noted [13, 23, 24]. A systematic review found that when mothers are supplemented to maintain levels above 40 ng/mL, PTB rates decrease [25]. Further, the risk of PTB is even higher with maternal levels under 30 ng/mL [9, 25, 26]. Thus, optimizing maternal vitamin D status may be associated with improved pregnancy outcomes. Prior studies have shown that a woman’s vitamin D status (as measured by 25(OH)D concentration) is stable throughout pregnancy unless there is a change in sunlight exposure, supplementation regimen, or dose [8, 27]. Accordingly, the specific timing of the 25(OH)D draw is unlikely to materially affect the measured level, although obtaining the value earlier in pregnancy naturally provides more opportunity for clinical intervention if deficiency is identified.

Although prior studies have suggested an association between maternal vitamin D status and the risk of preterm birth, results have varied across populations and study designs. Furthermore, limited data exist from large, racially diverse U.S. cohorts, particularly in the Southeastern United States, where vitamin D insufficiency is prevalent due to reduced time spent outdoors, lower sunlight exposure, and higher rates of melanin-associated reduction in cutaneous synthesis [14–16].

Our study addresses this gap by leveraging eight years of electronic health record data from a large academic medical center in South Carolina to examine the relationship between maternal 25(OH)D concentrations and gestational age at delivery. We hypothesized that lower maternal 25(OH)D concentrations would be associated with shorter gestational duration and an increased likelihood of preterm birth. Accordingly, we examined whether clinically obtained maternal 25(OH)D concentrations during pregnancy are associated with gestational age at delivery and preterm birth in a large, racially diverse cohort, with the goal of informing how existing vitamin D thresholds perform in real-world obstetric practice.

## Materials/subjects and methods

We conducted a retrospective chart review from January 2016 to March 2024 of women who delivered at the Medical University of South Carolina (MUSC) and had 25(OH)D measured during pregnancy. Using electronic health records (Epic) and ICD 10 and ICD 11 codes (see Supplementary Table 1), this yielded 15,506 subjects. Deidentified data were extracted, including information about gestational age, 25(OH)D concentration(s), and clinical and sociodemographic characteristics.

### Definition of preterm birth

Preterm birth was defined as delivery at <37 weeks and very preterm birth as <32, which were defined using established classifications endorsed by ACOG and WHO [1, 28].

### Assessment of vitamin D status

Serum 25-hydroxyvitamin D [25(OH)D] concentrations were obtained from the electronic health record (EHR). For each woman, the exposure of interest was defined as the first documented 25(OH)D measurement obtained during pregnancy and prior to delivery. This value was selected to minimize bias from repeat testing related to abnormal results, supplementation monitoring, or clinical events occurring later in pregnancy. Gestational age at the time of blood draw was calculated using standard obstetric dating and categorized by trimester. Although a subset of women had multiple 25(OH)D measurements during pregnancy, the timing of repeat testing was clinically driven and heterogeneous; therefore, subsequent measurements were not used to define the primary exposure.

### Measurement of 25(OH)D

Maternal vitamin D concentrations were measured from blood samples drawn from pregnant women receiving care at the Medical University of South Carolina’s clinical laboratory, using the first 25(OH)D concentration recorded. Serum 25-hydroxyvitamin D [25(OH)D] concentrations (Lab Code 535) were measured using a Liquid Chromatography-Tandem Mass Spectrometry (LC-MS/MS). If women had multiple 25(OH)D concentrations drawn during pregnancy, only the first concentration was recorded in the dataset.

### Conceptual framework

Conceptually, maternal vitamin D status during pregnancy was assessed at a single clinically obtained time point before delivery, with pregnancy duration influencing both the opportunity for vitamin D testing and the timing of delivery, necessitating trimester-based rather than time-to-delivery comparisons.

### Inclusion criteria

Women who had a 25(OH)D concentration drawn during pregnancy who delivered at the Medical University of South Carolina.

### Exclusion criteria

Mothers participating in vitamin D supplementation study in the experimental group were excluded from this analysis. Mothers with HIV or cancer were also excluded.

### Statistical analysis

All analyses were conducted using SAS version 9.4 (SAS Institute, Cary, NC). The primary outcome was preterm birth, defined as delivery before 37 completed weeks gestation. Early/very preterm birth, defined as delivery before 32 weeks gestation, and etiologic subtypes of preterm birth (spontaneous, medically indicated, preeclampsia/eclampsia–associated, and chorioamnionitis–associated) were examined as secondary, exploratory analyses to assess consistency across clinically relevant categories.

The primary exposure was maternal 25-hydroxyvitamin D [25(OH)D] concentration, analyzed both as a continuous variable and using a priori, clinically recognized thresholds (<10, <20, <30, and <40 ng/mL), corresponding to commonly used definitions of severe deficiency, deficiency, insufficiency, and sufficiency in pregnancy-related literature [13, 29]. Threshold-based analyses were conducted to enhance clinical interpretability and facilitate comparison with prior studies.

Multivariable logistic regression was used to estimate associations between maternal 25(OH)D concentration and preterm birth outcomes. Covariates were selected a priori based on a directed acyclic graph (DAG) informed by prior literature and biological plausibility. Race/ethnicity was identified as a key confounder and included in adjusted models. Other variables available in the electronic health record were evaluated but not included if they were considered potential mediators or did not demonstrate a consistent independent association with preterm birth. Results are presented as adjusted odds ratios (aORs) with 95% confidence intervals (CIs).

Relative risks (RRs) for preterm birth across 25(OH)D threshold categories were calculated using contingency tables and are presented as descriptive, unadjusted measures of absolute risk. Odds ratios derived from regression models are presented strictly as adjusted measures of association and were not interpreted as estimates of relative risk.

Because secondary outcomes represent overlapping and non-independent subsets of preterm birth, analyses beyond the primary outcome were interpreted as exploratory. Emphasis was placed on consistency in the direction and magnitude of associations rather than formal hypothesis testing across multiple endpoints. All statistical tests were two-sided, with a Type I error rate of 0.05.

**Fig. 1:**
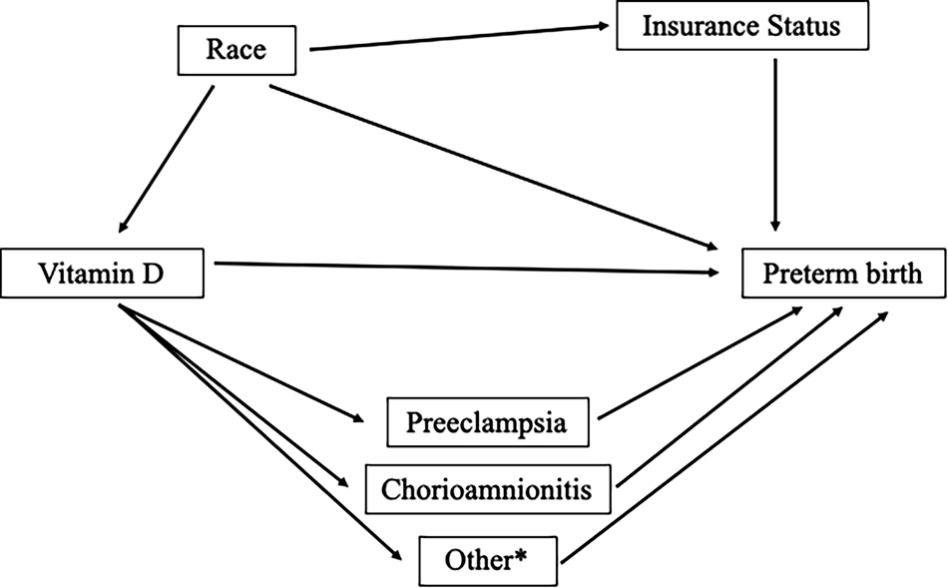
Directed Acyclic Graph. Directed acyclic graph illustrating the hypothesized relationship between maternal vitamin D status and preterm birth. Arrows indicate the presumed direction of influence between variables based on prior literature and biological plausibility.

As mentioned, covariate selection was guided by a DAG (Fig. 1) informed by prior literature and biological plausibility. The DAG represents hypothesized relationships among maternal 25(OH)D concentration, race/ethnicity, and preterm birth and was used to identify appropriate adjustment variables while avoiding control for potential mediators.

## Results

### Study population

**Fig. 2:**
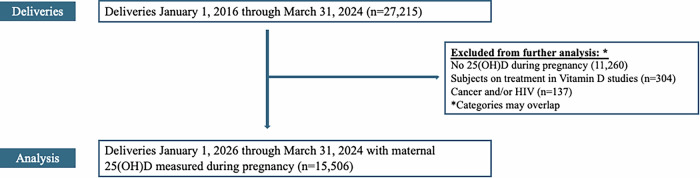
Flow diagram of study cohort.

Figure 2 illustrates the derivation of the analytic cohort. Of 27,215 deliveries identified during the study period, 15,506 women had at least one documented 25-hydroxyvitamin D [25(OH)D] measurement during pregnancy and were included in the final analysis. Reasons for exclusion are shown in Fig. 2. Table 1 provides the demographic characteristics of our study participants.

**Table 1:** Sociodemographic and clinical characteristics of participants*.

Category	Subgroup	*n*	%
Race/Ethnicity	Black American	4533	29.30%
	Hispanic	1385	8.90%
	White	8122	50.70%
	Other/unspecified	1466	9.50%
Maternal insurance status	Insured, private insurance	7583	48.90%
	Insured, government insurance	7321	47.20%
	Not insured or self-pay	79	0.50%
	Unknown insurance status	523	3.40%
Age (years)	17–20	924	6.00%
	21–25	2969	19.20%
	26–34	8458	54.60%
	35–46	3417	20.20%
	Mean (SD)	29.6 (5.6)	
Maternal comorbidities	Obesity	2668	17.10%
	Gestational diabetes	1161	7.40%
	Chronic diabetes	387	2.50%
	Pregnancy-induced hypertension	1405	9.00%
	Preeclampsia	697	4.50%
	Infection during pregnancy	832	5.30%
	Chorioamnionitis	372	2.40%
Obstetrics factors	Cesarean delivery	2554	16.40%
	Induction of labor	245	1.60%
	Preterm rupture of membranes	1083	6.90%
	Multigravida	1910	12.20%
	Small for gestational age	65	0.40%

*Characteristics are presented for the analytic cohort (women with measured 25(OH)D).

Of the 15,506 deliveries, 13,451 (86.9%) occurred at term (≥37 weeks), 1,652 (10.7%) were moderately preterm (32–36 weeks), and 385 (2.5%) were very preterm (<32 weeks); gestational age data were unavailable for 18 women (see Table 2). Mean maternal 25(OH)D concentrations were lower among women delivering preterm compared with those delivering at term, with the lowest concentrations observed among women delivering before 32 weeks (Table 2).

**Table 2:** Mean 25(OH)D (ng/mL) and gestational age.

	Mean ± SD (ng/mL)	Mean difference (compared to 37+ weeks)	95% CI	*p*-value
Overall	34.2 ± 17.0			
<32 weeks	26.2 ± 14.9	−8.56	−10.70 to −6.42	<0.0001
32–36 weeks	31.3 ± 17.8	−3.46	−4.54 to −2.38	<0.0001
37+ weeks	34.8 ± 17.3			

**Fig. 3:**
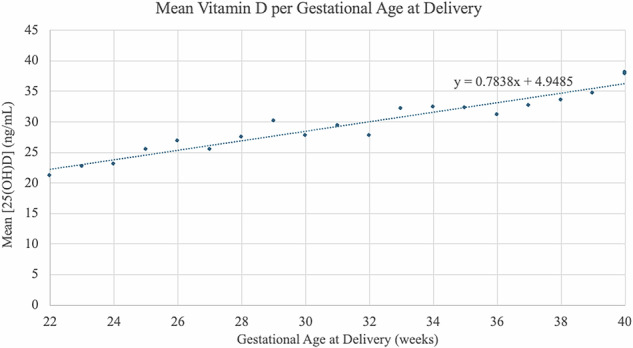
Distribution of Maternal 25(OH)D Concentrations by Gestational Age at Delivery. The x-axis represents gestational age at delivery (weeks), and the y-axis represents maternal 25(OH)D concentration (ng/mL). Scatterplot illustrating the distribution of maternal 25-hydroxyvitamin D [25(OH)D] concentrations by gestational age at delivery among women with at least one clinically obtained 25(OH)D measurement during pregnancy. Each point represents an individual pregnancy. The figure is descriptive and intended to provide visual context for the association between 25(OH)D concentration and gestational age at delivery; no causal inference is implied.

Overall, 6934 mothers (44.8%) had at least one measured 25(OH)D concentration below 30 ng/mL during pregnancy, and 10,257 (66.2%) had concentrations below 40 ng/mL. Figure 3 provides a descriptive visualization of the distribution of maternal 25(OH)D concentrations by gestational age at delivery.

The mean 25(OH)D concentrations were 40.8, 24.4, 33.8, and 28.5 ng/mL for White, Black, Hispanic, and other/unknown races, respectively. In multivariable logistic regression models adjusting for race/ethnicity, insurance status, and maternal age, higher maternal 25-hydroxyvitamin D concentrations were associated with lower odds of preterm birth <37 weeks and <32 weeks (see Table 3). Associations between sociodemographic covariates and preterm birth outcomes are shown in Table 3, with variation observed across race/ethnicity categories and gestational age thresholds.

**Table 3:** Multivariable logistic regression with adjusted odds ratios for preterm birth based on race, insurance status, and age.

	Birth < 37 weeks		Birth < 32 weeks	
	aOR (95% CI)	*p*-value	aOR (95% CI)	*p*-value
Black	1.59 (1.42–1.77)	<0.0001*	2.45 (1.92–3.14)	<0.0001*
Hispanic	0.75 (0.62–0.90)	0.0018*	1.55 (1.07–2.25)	0.0219*
Other race/ethnicity	1.44 (1.25–1.67)	<0.0001*	2.08 (1.52–2.86)	<0.0001*
Government insurance	1.32 (1.20–1.45)	<0.0001*	1.03 (0.83–1.26)	0.814
Self-pay	0.82 (0.41–1.63)	0.568	0.81 (0.20–3.36)	0.776
Maternal age 17–20 years	0.53 (0.43–0.65)	<0.0001*	0.45 (0.28–0.71)	0.0006*
Maternal age 21–25 years	0.63 (0.55–0.72)	<0.0001*	0.66 (0.50–0.88)	0.0039*
Maternal age 26–34 years	0.77 (0.69–0.86)	<0.0001*	0.72 (0.57–0.91)	0.0057*
[25(OH)D] (ng/mL)	0.992 (0.989–0.994)	<0.0001*	0.978 (0.971–0.985)	<0.0001*

*Stastitically significant.

## Discussion and conclusions

In this large retrospective cohort of more than 15,000 pregnancies at a single academic medical center, lower maternal 25-hydroxyvitamin D 25(OH)D concentrations were associated with earlier gestational age at delivery. Women delivering between 32 and 36 weeks had lower mean 25(OH)D concentrations than those delivering at term, and this difference was more pronounced among women delivering before 32 weeks. Collectively, these findings are consistent with prior studies reporting an inverse association between maternal vitamin D status and preterm birth and suggest that lower 25(OH)D concentrations are observed more frequently among women delivering preterm.

Nearly two-thirds of women in this cohort had at least one measured 25(OH)D concentration below 40 ng/mL, a threshold that has been proposed in some studies as optimal for maternal and fetal health [8, 13, 30]. Although the clinical significance of this threshold remains debated, the high prevalence of lower 25(OH)D concentrations in this population underscores the continued relevance of vitamin D status during pregnancy [8]. Prior observational studies and systematic reviews have reported lower rates of preterm birth among women with higher 25(OH)D concentrations [26, 31–35], providing context for the associations observed in this cohort.

Across predefined 25(OH)D categories, women with lower concentrations had higher proportions of preterm birth, a pattern that has been described previously in diverse populations [36, 37]. These findings align with reports by Kokkinari et al. and others demonstrating an association between maternal vitamin D deficiency and increased risk of delivery before both 37 and 32 weeks’ gestation [25, 26, 33–35]. A systematic review additionally found that maternal vitamin D deficiency is associated with birth before 37 weeks and before 32 weeks, further corroborating our findings [26]. While these analyses were descriptive and exploratory, the consistency of associations across gestational age thresholds supports continued investigation into vitamin D status as a potentially relevant factor in preterm birth risk.

Maternal 25(OH)D concentrations increased with advancing gestational age at delivery, a pattern that has been reported by other observational studies [32]. From a clinical perspective, even modest prolongation of gestation is associated with meaningful improvements in neonatal outcomes, highlighting why factors associated with gestational duration remain of interest. These findings do not establish causality but are compatible with the hypothesis that maternal vitamin D status may be linked to mechanisms influencing the timing of delivery [25, 35].

Mean maternal 25(OH)D concentrations remained relatively stable over the nine-year study period, suggesting no major secular changes in vitamin D status within this population. This stability likely reflects the absence of standardized screening or supplementation practices during the study years, consistent with the lack of uniform recommendations from major professional organizations [18, 38]. Together, these observations highlight ongoing variability in clinical approaches to vitamin D assessment during pregnancy.

In adjusted analyses, the inverse association between maternal 25(OH)D concentration and preterm birth persisted after accounting for race/ethnicity, insurance status, and maternal age [39–41]. Although heterogeneity in preterm birth risk across sociodemographic groups was observed [16, 39, 42], vitamin D status remained associated with gestational age at delivery across models. These findings suggest that maternal vitamin D status is not simply a surrogate for demographic or socioeconomic factors, although residual confounding cannot be excluded.

Several biological mechanisms have been proposed to explain a potential link between vitamin D and preterm birth [31, 33, 42, 43], including modulation of immune and inflammatory pathways, placental function, and fetal growth. Transcriptomic and mechanistic studies suggest that vitamin D insufficiency in early pregnancy may influence pathways relevant to parturition, providing biological plausibility for the observed associations. However, the extent to which these mechanisms operate in clinical populations remains an area for further study.

A substantial body of prior work provides important context for interpreting these findings. Over the past three decades, randomized supplementation trials, prospective cohort studies, and mechanistic investigations have collectively suggested that maternal vitamin D status may play a role in pregnancy outcomes, including preterm birth. While observational studies such as the present analysis cannot establish causality, the consistency of associations across study designs strengthens the biological plausibility of a contributory role for vitamin D in gestational duration.

Several studies have emphasized the importance of timing of vitamin D deficiency during pregnancy [44]. Early pregnancy 25(OH)D concentrations, particularly during the first trimester, appear to be more strongly associated with adverse pregnancy outcomes than concentrations measured later in gestation, and deficiencies early in pregnancy may have lasting effects even when corrected in the second or third trimester [19]. These findings suggest the existence of a critical window during which vitamin D status may be particularly relevant to placental development, immune regulation, and pathways involved in parturition. Informed by this literature, we prioritized the first clinically obtained 25(OH)D measurement during pregnancy as the exposure of interest, as later measurements may fall outside this biologically relevant window.

Although a 25(OH)D concentration of ~40 ng/mL has been proposed by some investigators as optimal for pregnancy outcomes, evidence for a universal threshold remains inconsistent across populations and study designs [13, 15, 26, 32]. Nevertheless, vitamin D’s established roles in calcium metabolism, skeletal development, and immune function [8–10, 26], together with emerging transcriptomic data demonstrating dysregulation of immune and inflammatory pathways in vitamin D insufficiency, provide plausible mechanisms linking low maternal vitamin D status to preterm birth [11, 13, 26, 32]. In addition, vitamin D deficiency has been associated with other pregnancy complications related to prematurity, including fetal growth restriction and small-for-gestational-age birth, suggesting broader relevance to perinatal health [9, 10].

Randomized trials of vitamin D supplementation have demonstrated that screening and supplementation can safely and effectively identify and correct deficiency during pregnancy, although trials have varied in dosing strategies, timing of intervention, and outcome definitions. Taken together, these data support continued investigation into whether optimizing maternal vitamin D status, particularly early in pregnancy, may influence gestational duration, while underscoring the need for well-designed prospective studies to clarify causal pathways, optimal thresholds, and populations most likely to benefit [13, 23, 24, 45].

This study has important limitations. Its retrospective design limits causal inference, and data on potential confounders such as maternal body mass index, diet, supplement use, and detailed socioeconomic measures were incomplete or unavailable. The timing of 25(OH)D measurement varied across pregnancy, although prior studies suggest that 25(OH)D concentrations remain relatively stable in the absence of major changes in supplementation or sun exposure. Additionally, vitamin D measurements were available only for women who underwent clinical testing, limiting generalizability to the broader obstetric population. Finally, because odds ratios do not directly estimate risk when outcomes are not rare, adjusted associations should be interpreted as measures of association rather than absolute risk.

Despite these limitations, this study contributes to the existing literature by providing data from a large, racially diverse cohort in the Southeastern United States, a region where vitamin D insufficiency remains common. The findings reinforce prior observations that lower maternal 25(OH)D concentrations are associated with earlier gestational age at delivery and highlight the need for continued research to clarify timing, thresholds, and potential clinical implications.

## Conclusions

In this large retrospective cohort, lower maternal 25-hydroxyvitamin D concentrations were associated with shorter gestational duration and higher odds of preterm birth. Although these findings do not establish causality, they contribute real-world evidence from a large, diverse U.S. population to a growing literature suggesting that maternal vitamin D status may be relevant to pregnancy outcomes. Further prospective studies and randomized trials are needed to determine whether optimizing vitamin D status during pregnancy can meaningfully reduce the risk of preterm birth and to identify populations most likely to benefit from targeted interventions.

## Supplementary information

Supplemental Materials

## Data Availability

The datasets generated during and/or analyzed during the current study are available from the corresponding author on reasonable request.
