# Supplementary material for: Maternal vitamin D status and preterm birth: an eight-year retrospective cohort study in the Southeastern United States
Source: J Perinatol. 2026 Jun 22;46(7):1300–6. doi: 10.1038/s41372-026-02757-z (PMC13423786; doi:10.1038/s41372-026-02757-z)
Supplement: Supplementary file 1 — Supplemental Materials [file 41372_2026_2757_MOESM1_ESM.docx]

***Supplemental Materials***

***Supplementary Table 1. ICD Codes of Excluded Participants***

| **Condition Category** | **ICD Codes Excluded** |
| --- | --- |
| **HIV/AIDS** | 042, B20 |
| **Cancer/Malignancy** | 642.1, 642.10, 642.11, 642.12, 642.13, 642.14, 043, 044, B21, B22, B23, B24, 140–165, 170–176, 179–199, 200–209, 225, 230–234, 237, 238.7, C00–C05, C07–C18, C19–C26, C30–C34, C37–C41, C43–C44, C46, C48, C50–C58, C60–C62, C64–C69, C71–C76, C77–C80, C81–C86, C90–C95, C4A, C7A, C7B, D00–D07, D09, D32–D33, D42–D47 |

***Supplementary Table 2. 25(OH)D Concentrations (ng/mL) per Year***

| **Year** | **Number of Deliveries** | **Average 25(OH)D (ng/mL)** | **Standard Deviation** | **Min25(OH)D (ng/mL)** | **Max 25(OH)D (ng/mL)** |
| --- | --- | --- | --- | --- | --- |
| 2016 | 970 | 33.1 | 14.7 | 4.4 | 88.5 |
| 2017 | 1,684 | 36.6 | 17.1 | 4.4 | 118.8 |
| 2018 | 1,293 | 37.1 | 18.8 | 4.7 | 128.9 |
| 2019 | 1,224 | 34.3 | 17.7 | 3.4 | 136 |
| 2020 | 1,254 | 34.5 | 18.5 | 3.5 | 117 |
| 2021 | 1,719 | 33.7 | 17.4 | 4.2 | 116.5 |
| 2022 | 2,917 | 32.7 | 17.4 | 4.1 | 134.7 |
| 2023 | 3,178 | 33.4 | 16.9 | 3.6 | 123.5 |
| 2024 | 1,267 | 34.9 | 16.9 | 4.9 | 140 |

To assess potential temporal trends in vitamin D status over the study period, we summarized the annual distribution of 25-hydroxyvitamin D [25(OH)D] concentrations for all deliveries from 2016 through 2024 (**Supplementary Table 2**). For each calendar year, we report the number of deliveries, mean 25(OH)D concentration, standard deviation, and minimum and maximum observed values. Across the nine-year period, mean annual 25(OH)D values remained relatively stable, ranging from approximately 32.7 ng/mL to 37.1 ng/mL.

***Supplementary Table 3. Relative Risk of Delivering Preterm Compared to Maternal 25(OH)D Thresholds***

| **25(OH)D (ng/mL)** | **% delivering <37 weeks** | **Relative Risk** | **95% CI** | **p-value** |
| --- | --- | --- | --- | --- |
| ≤10 | 22% | 1.72 | 1.49 - 2.00 | <0.001 |
| >10 | 12.70% |  |  |  |
| ≤20 | 18.87% | 1.64 | 1.51 - 1.79 | <0.001 |
| >20 | 11.46% |  |  |  |
| ≤30 | 16.37% | 1.55 | 1.43 - 1.68 | <0.001 |
| >30 | 10.54% |  |  |  |
| ≤40 | 14.63% | 1.43 | 1.30 - 1.56 | <0.001 |
| >40 | 10.26% |  |  |  |

As seen in **Supplementary** **Table 3**, we examined whether lower maternal 25(OH)D concentrations were associated with an increased risk of preterm delivery using predefined clinical thresholds. Among mothers with 25(OH)D <10 ng/mL, 22.0% delivered <37 weeks compared with 12.7% among those ≥10 ng/mL. Similarly, 18.9% of mothers with levels <20 ng/mL, 16.37% with <30 ng/mL, and 14.63% with <40 ng/mL delivered preterm. Corresponding relative risks increased in a stepwise manner with decreasing vitamin D levels: RR 1.73 (95% CI 1.49–2.00) for <10 ng/mL; RR 1.64 (95% CI 1.51–1.79) for <20 ng/mL; RR 1.55 (95% CI 1.43–1.68) for <30 ng/mL; and RR 1.43 (95% CI 1.30–1.56) for <40 ng/mL (all p < 0.001).

***Supplementary Table 4. Preterm Birth Rates and Maternal 25-Hydroxyvitamin D Concentrations by Race and Ethnicity***

| **Race/Ethnicity** | **N total** | **<32 weeks n (%)** | **<37 weeks n (%)** | **Mean 25(OH)D <32 weeks** | **Mean 25(OH)D in ≥32 weeks** | **p-value** | **Mean 25(OH)D <37 weeks** | **Mean 25(OH)D in ≥37 weeks** | **p-value** |
| --- | --- | --- | --- | --- | --- | --- | --- | --- | --- |
| White/Asian | 8,120 | 106 (1.31%) | 844 (10.39%) | 35.88 | 40.87 | 0.0016 | 38.89 | 41.02 | 0.0003 |
| Black | 4,532 | 201 (4.44%) | 828 (18.27%) | 22.08 | 24.55 | 0.0214 | 22.78 | 24.81 | 0.0004 |
| Hispanic | 1,385 | 30 (2.17%) | 122 (8.81%) | 27.22 | 33.99 | 0.0187 | 33.18 | 33.9 | 0.6269 |
| Other/Unknown | 1,451 | 48 (3.31%) | 243 (16.75%) | 21.82 | 28.6 | 0.0041 | 25.36 | 28.98 | 0.0014 |

**Supplementary Table 4** presents preterm birth rates at <32 weeks and <37 weeks of gestation and mean early pregnancy 25(OH)D concentrations stratified by race and ethnicity. Preterm birth rates are reported as number (percentage). Mean 25(OH)D concentrations (ng/mL) are shown separately for women delivering preterm and those delivering at ≥32 weeks or ≥37 weeks. Within each racial/ethnic group, differences in mean 25(OH)D concentrations between preterm and non-preterm deliveries were evaluated using two-sample *t* tests, and corresponding p-values are reported.
